# Genome-wide association studies of seedling quantitative trait loci against salt tolerance in wheat

**DOI:** 10.3389/fgene.2022.946869

**Published:** 2022-09-07

**Authors:** Rao Waqar Ahmad Khan, Rao Sohail Ahmad Khan, Faisal Saeed Awan, Ahmed Akrem, Arslan Iftikhar, Farhana Naureen Anwar, Hind A. S. Alzahrani, Hameed Alsamadany, Rana Khalid Iqbal

**Affiliations:** ^1^ Institute of Molecular Biology and Biotechnology, Bahauddin Zakariya University, Multan, Pakistan; ^2^ Centre of Agricultural Biochemistry and Biotechnology (CABB), University of Agriculture, Faisalabad, Pakistan; ^3^ Botany Division, Institute of Pure and Applied Biology, Bahauddin Zakariya University, Multan, Pakistan; ^4^ Department of Physiology, Faculty of Life Sciences, Government College University, Faisalabad, Pakistan; ^5^ Department of Pharmacy Practice, Bahauddin Zakariya University, Multan, Pakistan; ^6^ Department of Biology, College of Science, Imam Abdulrahman Bin Faisal University, Dammam, Saudi Arabia; ^7^ Department of Biological Sciences, Faculty of Science, King Abdul Aziz University, Jeddah, Saudi Arabia

**Keywords:** genome-wide, association mapping, wheat, salinity, seedlings

## Abstract

Salinity is one of the significant factors in decreasing wheat yield and quality. To counter this, it is necessary to develop salt-tolerant wheat varieties through conventional and advanced molecular techniques. The current study identified quantitative trait loci in response to salt stress among worldwide landraces and improved varieties of wheat at the seedling stage. A total of 125 landraces and wheat varieties were subjected to salt treatment (50, 100, and 150 mM) with control. Morphological seedling traits, i.e., shoot length, root length, and fresh and dry shoot and root weights for salinity tolerance were observed to assess salt tolerance and genetic analysis using SNP data through DArT-seq. The results showed that, at the seedling stage, 150 mM NaCl treatment decreased shoot length, root length, and fresh and dry weights of the shoot and root. The root length and dry root weight were the most affected traits at the seedling stage. Effective 4417 SNPs encompassing all the chromosomes of the wheat genome with marker density, i.e., 37%, fall in genome B, genome D (32%), and genome A (31%). Five loci were found on four chromosomes 6B, 6D, 7A, and 7D, showing strong associations with the root length, fresh shoot weight, fresh root weight, and dry root weight at the *p* < 0.03 significance level. The positive correlation was found among all morphological traits under study.

## Introduction

Crops grown in saline soils are always in constant threat of the salinity effect on their yield. Saline conditions in the soil do appear when excessive salts accumulate on the surface of the soil or in the plant’s root zone and are unable to leach down. Excessive use of salty water for irrigation and precipitation of the underground salts are the two major causes of salinity in the soil (Ali and Rab, 2017). High salt concentration in the root zone of the plant increases the osmotic pressure of the root cells, resulting in toxicity of ions in the cells. Saline stress causes a decrease in the water potential of cells and the unavailability of nutrients from the soil to the plant. This stress hampers the process of photosynthesis, transpiration, and metabolism, ultimately resulting in decreased plant growth and yield (Ali and Rab, 2017).

Wheat (*Triticum aestivum* L.) is a major staple crop, which is consumed mainly in Asia and 1/3rd population of the world. It is among the major cereal crops that show moderate tolerance to salt stress compared to rice and barley (Munns, James, and Läuchli, 2006). With the rapid development in DNA sequencing and its use in DNA marker identification, several quantitative trait loci (QTLs) have been identified for traits in wheat against salt tolerance, e.g., QTLs for yield traits (Eleuch et al., 2008; Xue et al., 2009; Fiaz et al., 2021), seedling and crop maturity (Lindsay et al., 2004; Quarrie et al., 2005; Zhao, Ma, and Ren, 2007; Genc et al., 2010), plant survival (Zhou et al., 2012), and salt exclusion in the shoot (Shavrukov et al., 2010). Generally, the traditional QTL mapping was found less sufficient in detecting genetic variation in wheat for salt tolerance (Shi et al., 2017).

The genome-wide association study (GWAS) emerged as a powerful tool, in which hundreds of individuals were genotyped, having a less genetic relationship. In this technique, the genotype data are associated with phenotype data on a trait of interest to identify significant marker-trait associations (Long et al., 2013; Turki et al., 2015; Sandhu et al., 2022) in field crops. The major sources of the erroneous connections are population structure and family relatedness/kinship, and these associations are avoided in the improved GWAS models by including the population structure and kinship matrix components. GWAS has been rapidly used since the initial association mapping in wheat for analyzing the genetic basis of several significant characteristics (Sandhu et al., 2021). The implementation of this approach is hampered by the fact that the majority of QTLs discovered using the GWAS are population-specific, have a small effect, and are difficult to estimate precisely. Nowadays, various types of molecular markers such as DArT and SNPs are commonly used by molecular breeders. The advancement in next-generation sequencing (NGS), higher genome coverage, and continuous reduction in the cost of genome sequencing, automated data acquisition, and analysis make SNPs a marker of choice for association studies. These markers are playing a significant role to speed up the process of marker-assisted selection (MAS). Genetic markers are also used to dissect the linkage disequilibrium (LD), population structure, and genome-wide marker-trait association among various polygenic traits of interest (Metzker, 2010). Salt-tolerant genes and QTLs for both abiotic stresses are identified in wheat and barley. They suggested that drought and salinity are the major abiotic stresses that threaten food security in the world (Nevo and Chen, 2010).

Wheat genotypes were screened out against salinity using different experiments, e.g., hydroponic, greenhouse, and field (Xu et al., 2013; Oyiga et al., 2018; Hussain et al., 2020). Many phenotypic traits for salt tolerance had, previously, been searched against NaCl at several concentrations (Ma et al., 2007; Genc et al., 2010; Oyiga et al., 2018). This study will be a fine attempt to explore the power of GWAS and SNP markers to dissect the genetic bases of salt stress in the wheat crop at the seedling stage.

## Materials and methods

### Plant materials

To study the effect of salt stress at the seedling stage in wheat, the experiment was conducted using a completely randomized design (CRD) with three replications. In this experiment, 125 worldwide landraces and improved varieties of wheat (Supplementary Table S1) were evaluated twice at three level of salinity i.e., 0, 50, 100, and 150 mM NaCl).

### Experimental design

The seedling stage experiment was conducted in a glasshouse using plastic bags filled with soil. Saline water was applied for 40 days. All plants were harvested with the care that roots were not damaged. Plants were removed from plastic bags in running water, and excessive water was removed with tissue papers. Shoot length, root length, and fresh weights of shoot and root were taken. Dry weights of shoots and roots were recorded after drying all plants in the oven for 48 h.

### Phenotypic trait measurement

1) Shoot length (SL) and root length (RL): it was recorded with a scale at 6 weeks after sowing.
2) Fresh shoot weight (FSW) and fresh root weight (FRW): shoot and root weights were measured on an electrical balance just after harvesting.
3) Dry shoot weight (DSW) and dry root weight (DRW): the shoot and root were placed in the oven for 48 h at 60°C. Dry weight was measured on an electrical balance.
4) Percentage of increase or decrease: Percentage of increase or decrease for each characteristic was calculated by the difference (increase or decrease) between the two numbers (comparing), then dividing the increase or decrease by the original number, and multiplying the answer by 100.

$$C=\left(\frac{X2-X1\;}{X1\;}\right)X\;100.$$

X1 = initial value, and X2 = final value of the characteristic.

### Genotyping

DNA was extracted from leaves of wheat plants and sown under salt treatments for marker analysis. In short, the cetyltrimethylammonium bromide (CTAB) method was adopted for the extraction of the genomic DNA from leaves which were collected, transferred in liquid nitrogen at the time of sampling, and stored at F02D80°C. DNA was quantified with a Nano-Drop 8000 spectrophotometer (V.2.1.0). The DNA collected was genotypically characterized through the DArTseq™ technology (http://www.diversityarrays.com/dart-application-dartseq) of the Genetic Analysis Service for Agriculture (SAGA) service unit at the CIMMYT headquarters (Texcoco, Mexico). These SNPs were further assigned chromosomes, orders, and genetic distances, according to the 100K marker DArT-seq consensus map available at the Diversity Arrays Technology Pty Ltd. (DArT) (http://wwwdiversityarrays.com/sequence-maps).

### Statistical analysis

Analysis of variance (ANOVA) was calculated by Prism (version 9). Data for the SNP density plot, phenotypic histograms, Manhattan plot, and correlation plot were visualized in RStudio software. The structure of the population was determined using STRUCTURE (v.2.3.4.) (Pritchard, Stephens and Donnelly, 2000) based on an admixture model as in the model, the K-values ranged from 2 to 9 with five independent runs, the burn-in period was set at 100,000, and Markov chain Monte Carlo (MCMC) repetitions after burn-in were set at 100,000. The STRUCTURE HARVESTER (http://taylor0.biology.ucla.edu/structureHarvester/) was used to extract and analyze the results of the structure for an estimate of the optimal value of K using the delta (K) method. Genome-wide association analysis on phenotypic data was analyzed by using a mixed linear model (MLM) through TASSEL v 5.2.43 (Bradbury et al., 2007). The MLM can be represented (Sandhu et al., 2021) as

Y=SNP + Q[PCs] + Kinship + e,

where Y is a matrix of phenotypic information, SNP represents the matrix of markers, Q represents the population structure, and Kinship represents the relationship matrix between the individuals included in the model. SNP and Q are set as fixed effects, while kinship is a random effect in the model (Yu et al., 2006).

## Results

### Phenotypic correlations and ANOVA

**FIGURE 1:**
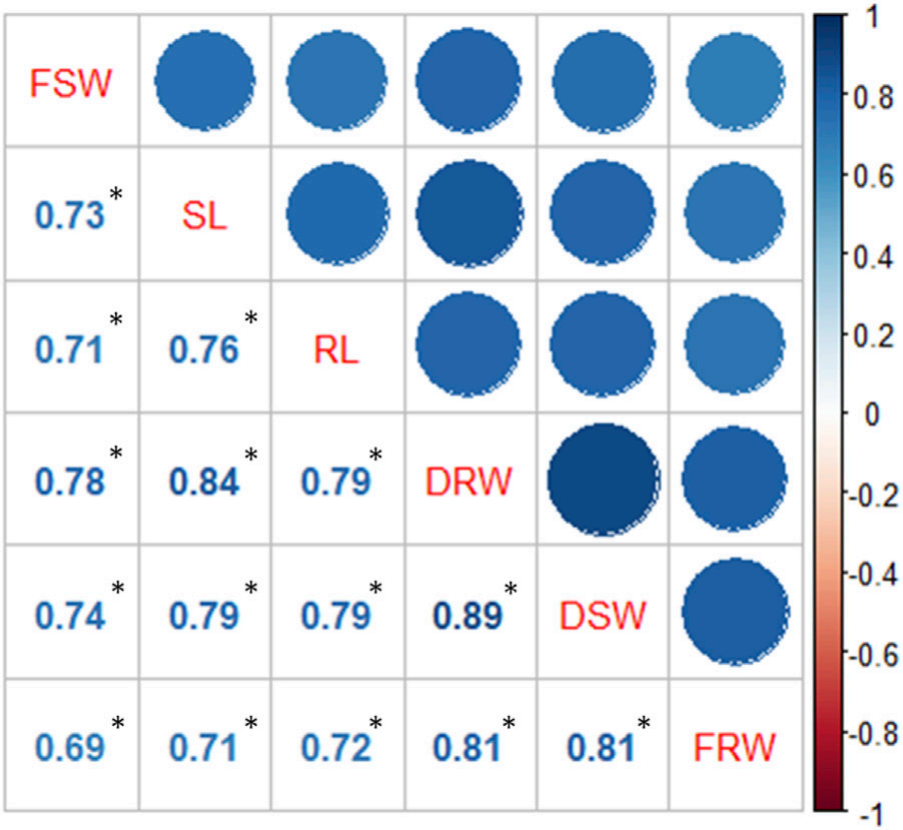
Correlation among seedling-related traits.

Treatments are highly significant, indicating the negative impact of salt on seedling-related traits (Table 1 and 2). Varieties and their interaction with salt treatments showed a high degree of variation for SL, RL, and FSW and were non-significant for FRW, DSW, and DRW (Table 1; Supplementary Figure S1, S2). It was observed that seedling-related traits under study were decreased when plants were exposed to high salts. A maximum percentage decrease (44.59%) was observed in RDW, followed by RL (40.15%) and FSW (38.28%) (Table 2), while the minimum was observed in FRW (21.45%). Strong significant correlations were found among all seedling-related traits (Figure 1, Supplementary Table S2).

**TABLE 1:** Mean square calculated through ANOVA for wheat genotypes and salinity treatments.

SOV	df	Shoot length (g)	Root length (g)	Fresh shoot weight (g)	Fresh root weight (g)	Dry shoot weight (g)	Dry root weight (g)
Treatment	3	2468.27***	1895.95***	1028.53***	249.34***	6.672***	7.717***
Variety	124	7.33***	5.23***	2.39***	0.149^ns^	0.017 ^ns^	0.0007 ^ns^
Treatment x Variety	372	1.85***	1.76***	1.93***	0.69 ^ns^	0.0016 ^ns^	0.0007 ^ns^
Error	1000	0.85	0.67	0.66	0.17	0.0018	0.0008

*significant at <0.05, **significant <0.01, and ***significant <0.001.

**TABLE 2:** Range, mean, and percentage change in seedling traits of 125 genotypes under control (0 mM) and salinity (150 mM).

Trait	Normal (0 mM)		Salt treatment (150 mM)		Percentage increase (+) or decrease (–)
	Range	Mean	Range	Mean	
Shoot length (cm)	20–23	21.35 ± 1.04	13–19	15.55 ± 1.67	−24.16
Root length (cm)	10–14	11.88 ± 1.2	5.00–8.67	7.11 ± 1.14	−40.15
Shoot fresh weight (g)	7.59–11.37	9.77 ± 1.14	4.67–7.74	6.03 ± 0.98	−38.28
Root fresh weight (g)	8.51–9.47	8.95 ± 0.40	6.43–7.58	7.03 ± 0.45	−21.45
Shoot dry weight (g)	0.793–0.886	0.83 ± 0.04	0.463–0.577	0.52 ± 0.05	−37.34
Root dry weight(g)	0.720–0.786	0.74 ± 0.04	0.40–0.475	0.41 ± 0.07	−44.59

### SNP density on the genome

**FIGURE 2:**
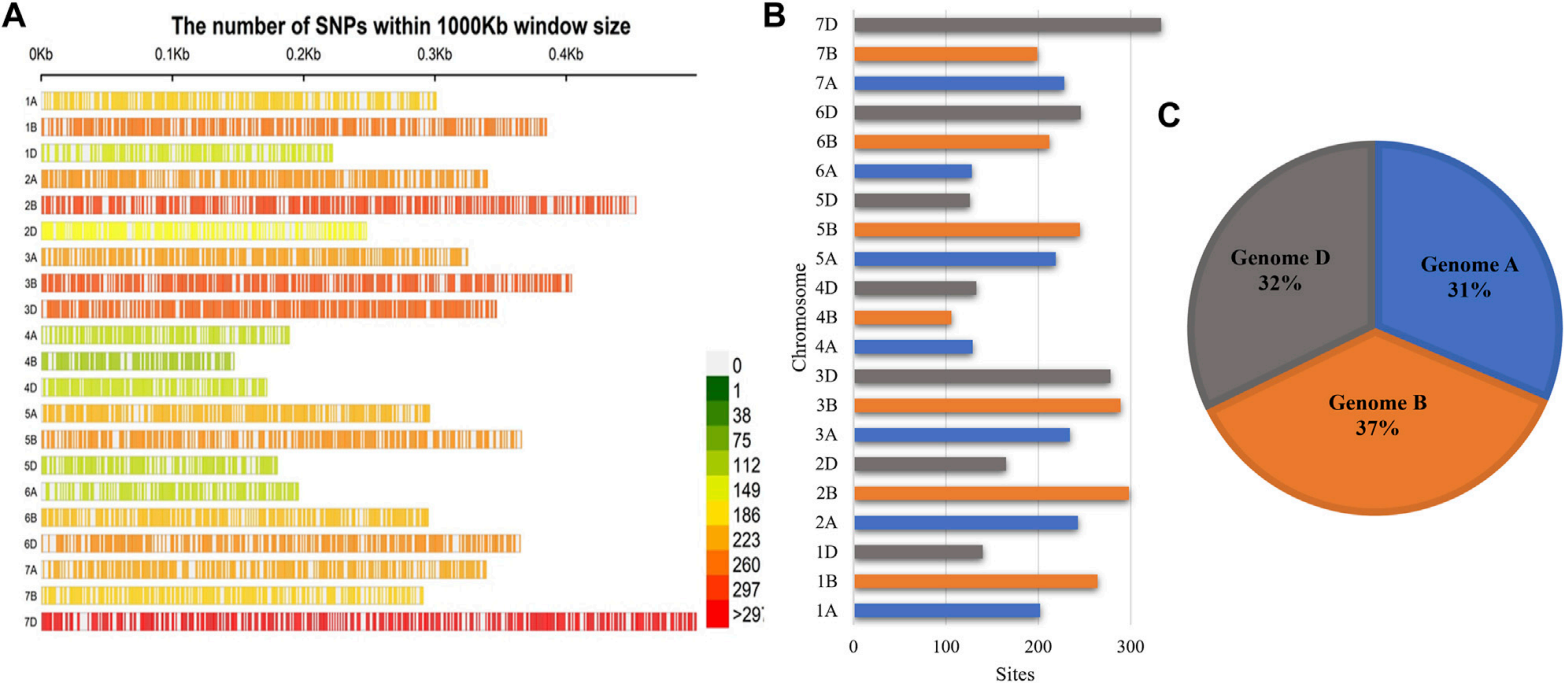
SNP density plot of the **(A)** SNPs mapped to the wheat genome in wheat lines; **(B)** the number of SNP sites present on each chromosome; **(C)** genome-wise distribution of SNPs.

A total of 4417 SNPs were found encircling all chromosomes of the wheat genome. Chromosomes varied in their length, and different numbers of SNPs were mapped on them. A minimum of 106 SNPs were observed at chromosome 4B, and a maximum of 333 SNPs were observed at chromosome 7D with a mean of 208.19 SNPs per chromosome. The chromosomal length varied between 0.148 cM (chromosome 4B) and 0.491 cM (chromosome 7D) (Figures 2A,B). The marker density was also not uniform among genome-like maximum markers; about 37% fall in genome B, followed by genome D (32%) and genome A (31%) (Figure 2C, Supplementary Table S3).

### Analysis of the population structure

**FIGURE 3:**
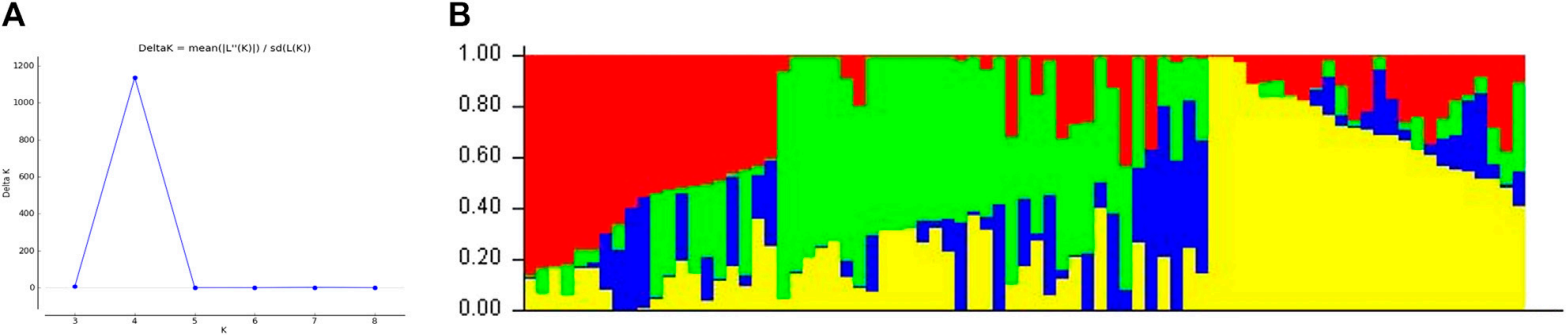
Determination of the **(A)** optimal value of K = 4 and **(B)** population structure of 79 wheat genotypes using DArT-seq SNP markers.

To reduce the possibility of unauthentic associations, all loci were selected to analyze the population structure of wheat varieties. The structure result at K = 4 found the best separator, which provides the highest delta k (∆k) value (Figure 3). Structure results divided the population into four sub-groups, and the overlapping phenomenon occurs between sub-groups because of wheat breeding material in the current study.

### Marker-trait associations

**FIGURE 4:**
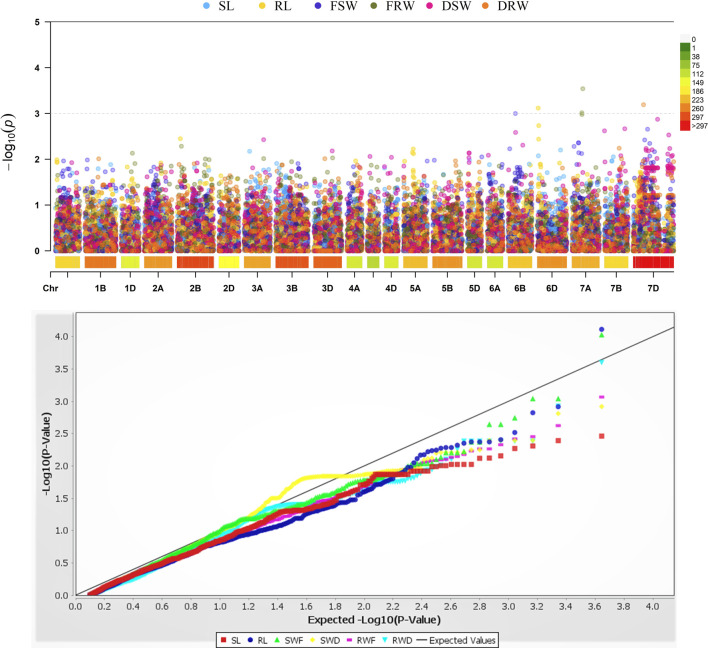
Genome-wide association analysis of seedling-related trait loci in the A, B, and D genomes of wheat. SL: shoot length; RL: root length; FSW: fresh shoot weight; FRW: fresh root weight; DSW: dry shoot weight; DRW: dry root weight.

It was found that five different loci exist on four different chromosomes 6B, 6D, 7A, and 7D, and they showed strong associations with the RL, FSW, FRW, and DRW (Figure 4; Table 3). These significant loci were associated (at *p* < 0.03 significance level) with R^2^, ranging between 0.14 and 0.22 for FSW and FRW, respectively (Table 3). The marker RS#1008453 was strongly associated with RL, present at chromosome 6B (Figure 4), while the marker RS#1067078 on chromosome 6D was associated with FSW (R2 = 0.14). Chromosome 7A has two marker sites, i.e., RS#1100610 and RS#2255164 associated with FRW, and explained 0.22 and 0.17 of variation, respectively. A marker (RS#1074330) on the 7D chromosome associated with DRW (R^2^ = 0.16) affects the dry root weight (Figure 3; Table 3). For the seedling growth stage, no marker was found to be significantly correlated with SL and DSW.

**TABLE 3:** Five trait loci significantly associated with salt-related seedling traits.

	Marker RS#	Chromosome	–log10 p value	R2
Root length (RL)	1008453	6B	0.000542	0.19
Fresh shoot weight (FSW)	1067078	6D	0.000235	0.14
Fresh root weight (FRW)	1100610	7A	0.000144	0.22
	2255164	7A	0.000603	0.17
Dry root weight (DRW)	1074330	7D	0.000609	0.16

R2 indicates the variation explained by the marker.

### Linkage disequilibrium

**FIGURE 5:**
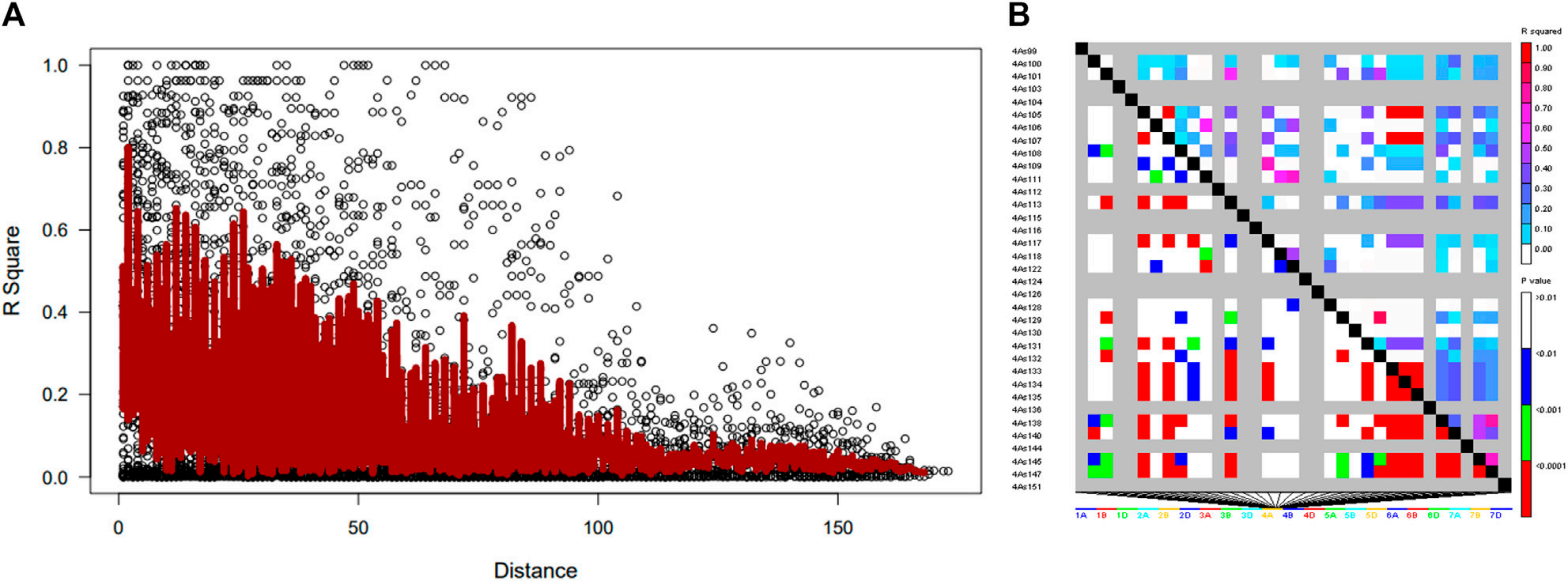
Linkage disequilibrium (LD)-measured **(A)** R2 plotted vs. the physical map (bp) between pairs of SNP markers in wheat genotype and **(B)** LD decay on chromosome 4.

The distribution of LD based on 4417 SNP markers showed extensive LD decay, as in the entire marker population. The range of linkage disequilibrium revealed elevated LD measures (average R^2^ = 0.5) over ranges as long as 200 Kb (Figure 5). The depreciation rate of LD was very slow and decayed to as low as R^2^ = 0.18. This indicates that pinpointing candidate genes in such a long-range LD is a tedious task and results in an exhaustive list. The overall LD decay in wheat lines was relatively low as it passes 100 Kb, and a few markers showed R^2^ ≥ 0.8. In total, 4417 SNP markers showed complete LD (R^2^ = 1), although a huge LD block was observed on chromosome 4.

## Discussion

Indicators used to evaluate the salt tolerance of plant germination include the seed germination rate, shoot length, and root length, so salt tolerance at the germination and seedling stage is very important. In the current study, seedling-related traits (shoot and root length (SL and RL) and dry and fresh weights of the shoot and root (FSW, FRW, DSW, and DRW) of 125 wheat accessions under salt stress demonstrated variation. Morphologically, germplasm showed a significant decrease in all traits measured at the seedling level. It is observed that fresh and dry weights of the root and shoot were decreased at high salt levels. The behavior of the root and shoot showed separate responses when the plant was subjected to salt stress (Lin et al., 2004). Shoots largely contain photosynthetic biomass including palisade mesophyll cells, thick cell wall, and epidermal layers, which do not degrade completely upon drying. On the other hand, roots largely comprise vascular bundles containing xylem and phloem with large spaces, and upon drying, they lose greater biomass; hence, differences between fresh and dry weights of roots are greater than those in case shoots. Generally decrease in the root length is considered to be a strong indicator of salt stress, but it was observed that alongside root length, the root dry weight may also be used as one of the key selection criteria for screening wheat lines against salinity, which is ultimately used in breeding programs. However, the concluded results are not always reliable due to different response patterns of some genotypes, which showed tolerance at the seedling stage but were unable to grow under continuous stress (Turki et al., 2015). The phenotypic positive correlation was found to be significant between seedling traits, which indicates the salt tolerance in wheat is not influenced (Mano and Takeda, 1997; Munns and Tester, 2008).

In wheat, numerous loci for salt tolerance have been identified in field- and hydroponic-based experiments against yield traits. There is no genome-wide study on wheat seedlings, especially keeping the shoot length, root length, and dry and fresh weights of the shoot and root as tolerance indicators for the association study. However, this phenomenon has also been reported in some crops, e.g., rice (Shi et al., 2017; Batayeva et al., 2018; Islam et al., 2022), flax (Li et al., 2022), *Camelina* (Luo, Szczepanek, and Abdel-Haleem, 2020), and barley (Ahmadi-Ochtapeh et al., 2015).

In the current study, it was found that the marker RS#1008453 is present at chromosome 6B for root length and the marker RS#1067078 for fresh shoot weight, located at the 6D chromosome. The loci RS#1100610 and RS#2255164 on chromosome 7A are associated with fresh root weight. Ma et., al (2007) found the locus Qpdws-2A.2/Qsfws-2A.1 for plant biomass in wheat. RS#1074330 was present at chromosome 7D for dry root weight. Liu et al. (2018) identified gwm251 on chromosome 4B as it was associated with fresh root weight at the seedling stage which was near QTL QTdw-4B controlling the total dry weight, as explored by Xu et al., 2013. Numbers of QTL for salinity tolerance in the various mapped populations of wheat have been detected for yield contributing traits (Ma et al., 2007; Genc et al., 2010; Xu et al., 2013; Ghaedrahmati et al., 2014). Association studies emerged as an efficient technique for detecting QTLs for desired traits in the same population at the same time. This technique creates opportunities for breeders for marker-assisted breeding (Liu et al., 2018). Five identified QTL regions, reported in the current study, have not been previously detected and could be used in future efforts to achieve a better plant selection against salt tolerance at the early stage (Zhu et al., 2008; Zhang et al., 2010, 2012). However, larger-scale multilocation field research would be necessary to confirm those identified SNPs.

## Conclusion

Genome-wide association studies provide an effective way to capture superior alleles that were not explored by conventional breeding methods. Introgression of these alleles into breeding germplasm supports breeders in using marker information in developing new varieties. In this study, we evaluated the salt tolerance of wheat accessions at the seedling stage and screened salt-tolerant germplasms. The salt stress significantly reduced the fresh and dry weights of the shoot and root. The 4417 SNPs were detected from the GWAS of salt tolerance-related traits of wheat accessions during the seedling stage. These SNPs were distributed as 31, 37, and 32% in three genomes A, B, and D, respectively. Important loci for important traits were found on chromosomes 6B, 6D, 7A, and 7D. Information about salt-tolerant loci is very important in improving and developing salt-tolerant wheat genotypes. It is important to have information about salt-tolerant loci in improving and developing salt-tolerant genetic breeding material using marker-assisted selection. However, to examine the genotype and environment interaction and to confirm those found SNPs, larger-scale multilocation field experiments in subsequent multiple years would be required.

## Data Availability

The original contributions presented in the study are included in the article/Supplementary Material; further inquiries can be directed to the corresponding author.
