# Supplementary material for: Genome-wide association studies of seedling quantitative trait loci against salt tolerance in wheat
Source: Front Genet. 2022 Sep 7;13:946869. doi: 10.3389/fgene.2022.946869 (PMC9492296; doi:10.3389/fgene.2022.946869)
Supplement: Supplementary file 1 [file Table1.DOCX]

Supplementary Table 1: List of wheat varieties/lines used for salinity study

| Code | Variety/Line | Code | Variety/Line | Code | Variety/Line |
| --- | --- | --- | --- | --- | --- |
| V1 | FAREED-06 | V44 | SHARP/3/PRL/SARA//TSI/VEE#5/5/VEE/LIRA//BOW/3/BCN/4/KAUZ | V87 | V-12253 |
| V2 | MIRAJ-08 | V45 | DOLLARBIRD | V88 | V-11160 |
| V3 | PASINA 90 | V46 | KIRITATI | V89 | 12266 |
| V4 | SA-42 | V47 | PFAU/WEAVER*2// | V90 | V-13005 |
| V5 | SATLUJ-86 | V48 | PGO/SERI//BAV92 | V91 | V-13016 |
| V6 | SHAHKAR 95 | V49 | NING MAI-50 | V92 | V-12130 |
| V7 | NACOZARI F-76 | V50 | CROC_1/AE.SQUARROSA (205)//FCT/3/PASTOR | V93 | V-12057 |
| V8 | WH-542 | V51 | HD 2169/C591//PBW343 | V94 | V-13241 |
| V9 | HOOSAM-3 | V52 | AS2002/WL711//SHAFAQ | V95 | V-12066 |
| V10 | SAAR | V53 | INQ91/YR-31 | V96 | V-13270 |
| V11 | CHAM-4 | V54 | V-04179/T7 (T.sphaerococcum) | V97 | 122557 |
| V12 | CHILERO=CHIL'S' | V55 | V-04179/T7 (T.sphaerococcum) –drought | V98 | 12BT012 |
| V13 | FRONTANA | V56 | WBLLI*2/VIVITSI/3/T.DICOCCOMP194624/AE.SQ(409)//BCN/4/WBLL1*2/ | V99 | 12C027 |
| V14 | HARTOG=HTG.(PAVON) | V57 | WBLLI*2/VIVITSI/3/T.DICOCCOMP194624/AE.SQ(409)//BCN/4/WBLL1*2/ | V100 | 10FJ21 |
| V15 | PARULA=PRL | V58 | TOBA97/PASTOR*2// | V101 | TW /424 |
| V16 | NING-8319 | V59 | T.SPELTA P1348764//INQ.91*2/TUKORU/3/WBLL1*2/TUKURU | V102 | MSW |
| V17 | HARRIER 17.B | V60 | V-11179 | V103 | NR-429 |
| V18 | PB81//F3.71/TRM/3/BULBUL | V61 | V-11186 | V104 | NR-449 |
| V19 | PB-96/87094//MH-97 | V62 | MUNAL #1 | V105 | Triticumpyrum (V-2) |
| V20 | WL 711/CROW “S”//ALD #1 / CMH77A.917/3/HI 666/PVN ‘S’ | V63 | TACUPETO F2001/BRAMBLING// | V106 | 088200 (mono tiller early maturity with less lodging) |
| V21 | KANZ*4/KS85-8-4/5/2*FRET2*2/4/SNI/ | V64 | ATTILA/3*BCN//BAV92/3/TILHI/5/BAV92/3/PRL/SARA//TSI/VEE#5/4/CROC_1/AE.SQUARROSA (224)//2*OPATA | V107 | TWS-12464 |
| V22 | CROC-1/AE.SQ(224)//OPATA/3/FLAG-7 | V65 | FRET2/KUKUNA//FRET2/3/PARUS/5/FRET2*2/4/SNI/TRAP#1/3/KAUZ*2/TRAP//KAUZ | V108 | NR-487 |
| V23 | SERI.1B*2/3/KAUZ*2/ | V66 | TRCH//PRINIA/PASTOR | V109 | 14C036 |
| V24 | 122526 | V67 | SOKOLL*2/TROST | V110 | NIBGE GANDUM N |
| V25 | Pak-2013 | V68 | LYP-73 | V111 | 14170 |
| V26 | 13248 | V69 | YECORA-70 Sr2 | V112 | morroco |
| V27 | Long grain | V70 | NR 388 | V113 | 10821 |
| V28 | NSW-14 | V71 | NR-371 | V114 | 11526 |
| V29 | V-12266 | V72 | NR-403 | V115 | Pasban90 |
| V30 | V-02192 | V73 | 76377 | V116 | Chakwal86 |
| V31 | V-04181 | V74 | 99108 | V117 | LU26s |
| V32 | V-04048 | V75 | V-11365 | V118 | 10849 |
| V33 | V-05115 | V76 | 11B2049 | V119 | Kohistan-97 |
| V34 | V-06129 | V77 | 11BT004 | V120 | 11380 |
| V35 | V-056132 | V78 | V-12284 | V121 | 11287 |
| V36 | TW69019 | V79 | NW-10-1111-7 | V122 | 11386 |
| V37 | V-06018 | V80 | V-11046 | V123 | 10813 |
| V38 | KIRITATI/4/2*SERI.1B*2/3/KAUZ*2/BOW//KAUZ | V81 | NR 411 | V124 | Chakwal97 |
| V39 | WHEAR/VIVITSI//WHEAR | V82 | V-11001 | V125 | 11464 |
| V40 | WHEAR/CHAPIO//WHEAR | V83 | 12292 |  |  |
| V41 | INQALAB91*2/KUKUNA// | V84 | D67.2/PARANA 66.270//AE.SQ (320)/3/CUNNINGHAM/4/VORB |  |  |
| V42 | SUNCO//TNMU/TUI | V85 | ATTILA*2/PBW65*2// |  |  |
| V43 | SUNCO//TNMU/TUI | V86 | PFAU/SERI.1B//AMAD/3/ |  |  |
