# Supplementary figures and images for: Genome-wide association studies of seedling quantitative trait loci against salt tolerance in wheat
Source: Front Genet. 2022 Sep 7;13:946869. doi: 10.3389/fgene.2022.946869 (PMC9492296; doi:10.3389/fgene.2022.946869)

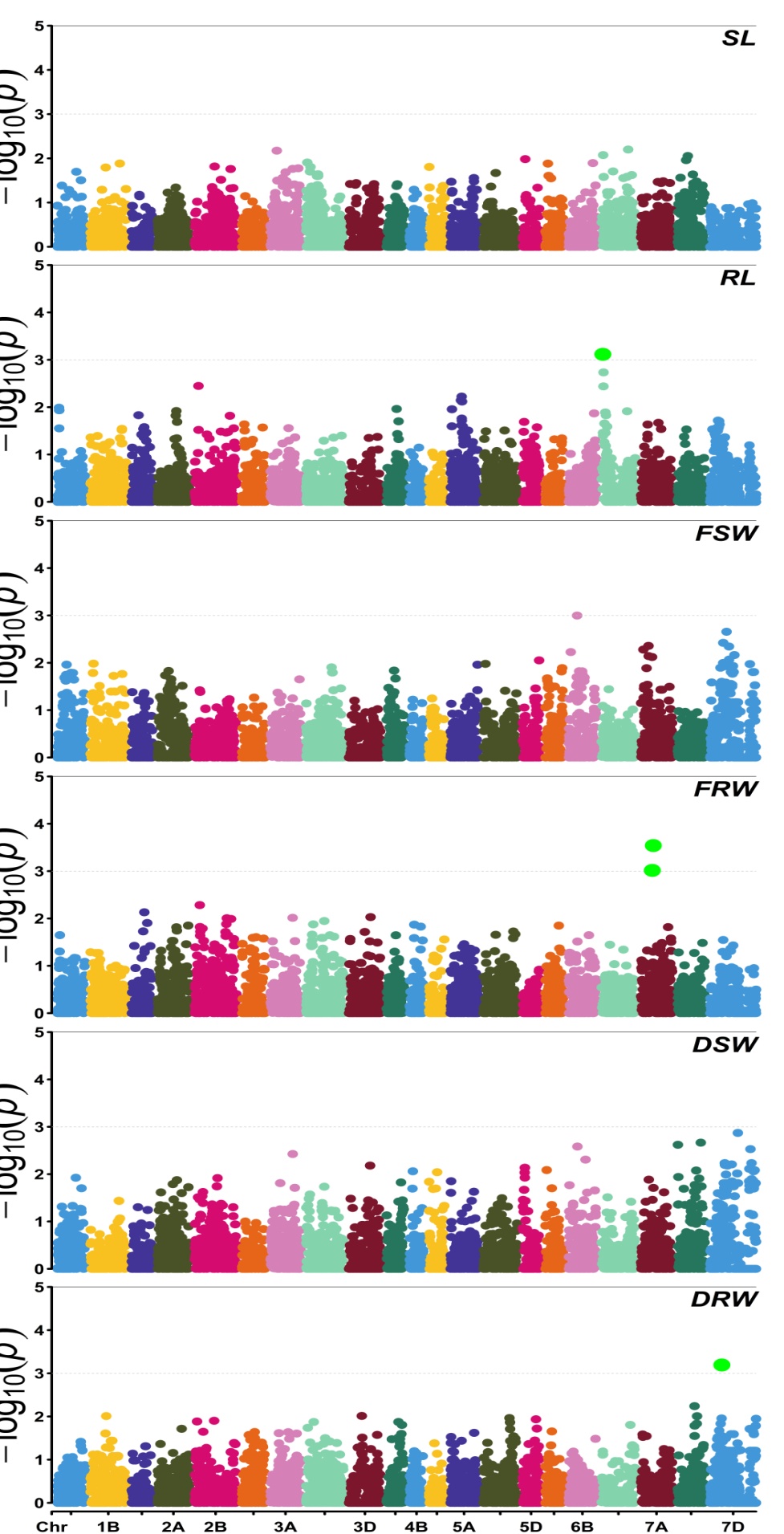


Supplementary Figure 3: Manhattan Plot on each seedling traits

Supplement: Supplementary file 2 [file DataSheet3.docx]

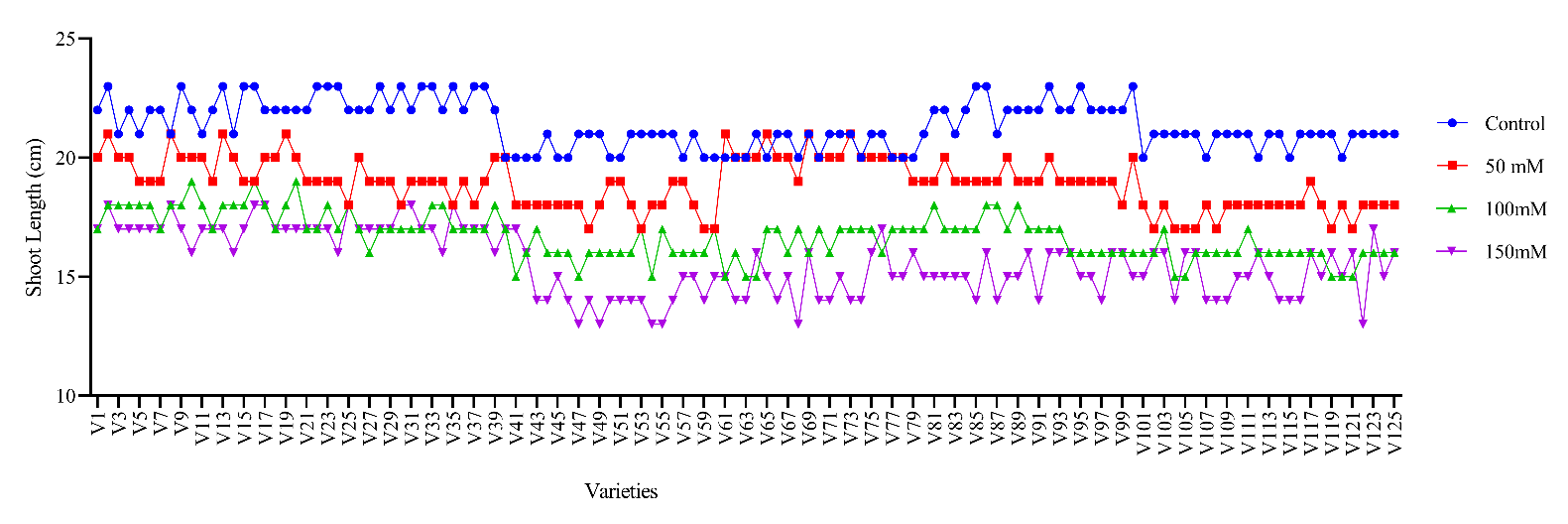


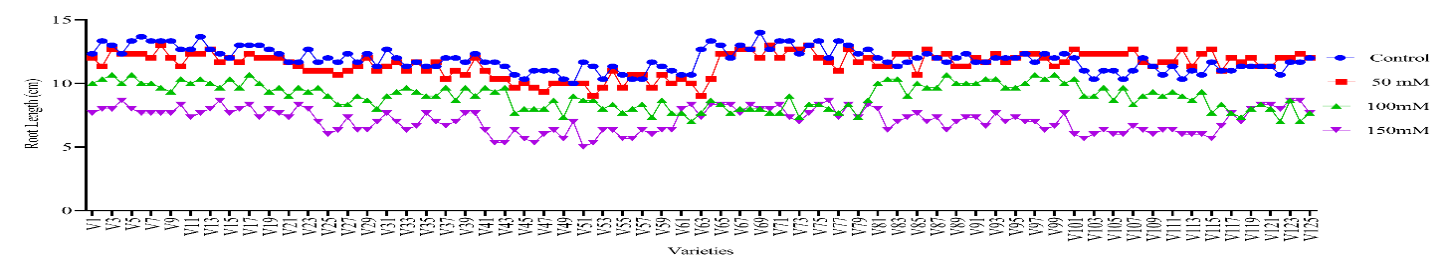


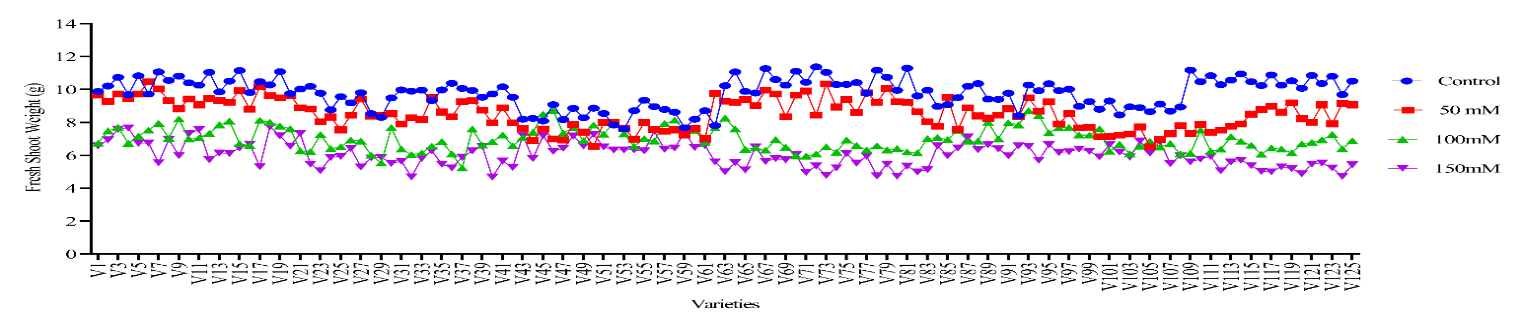


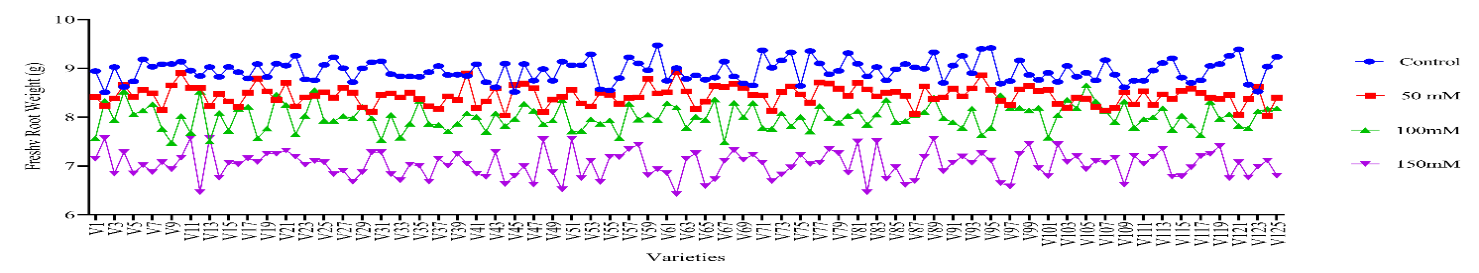


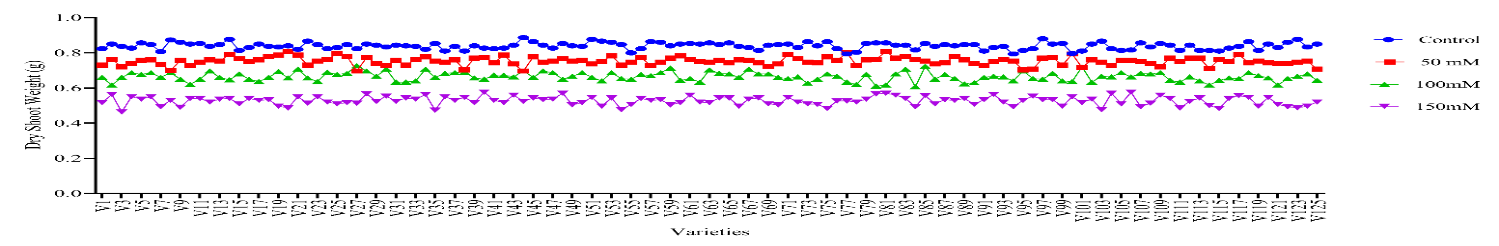


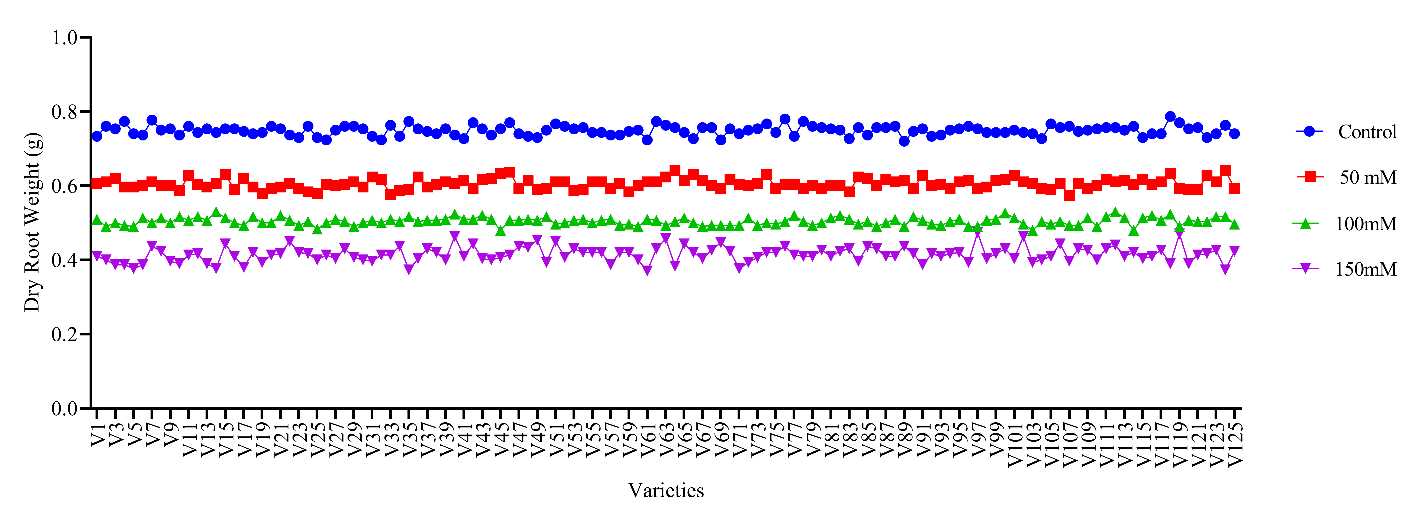


Supplementary Figure 2: Response of wheat varieties against salinity

Supplement: Supplementary file 5 [file DataSheet2.docx]
