# Supplementary material for: Genome-wide association studies of seedling quantitative trait loci against salt tolerance in wheat
Source: Front Genet. 2022 Sep 7;13:946869. doi: 10.3389/fgene.2022.946869 (PMC9492296; doi:10.3389/fgene.2022.946869)
Supplement: Supplementary file 3 [file Table2.DOCX]

Supplementary Table 2: Correlation among seedling traits

|  | **SL** | **RL** | **SW (F)** | **RW (F)** | **SW (D)** | **RW (D)** |
| --- | --- | --- | --- | --- | --- | --- |
| **Sl** | - |  |  |  |  |  |
| **RL** | 0.765* | - |  |  |  |  |
| **SW (F)** | 0.741* | 0.706* | - |  |  |  |
| **RW (F)** | 0.717* | 0.730* | 0.686* | - |  |  |
| **SW (D)** | 0.792* | 0.793* | 0.749* | 0.816* | - |  |
| **RW (D)** | 0.835* | 0.785* | 0.789* | 0.810* | 0.896* | - |

.

*significant
