# Supplementary material for: Genome-wide association studies of seedling quantitative trait loci against salt tolerance in wheat
Source: Front Genet. 2022 Sep 7;13:946869. doi: 10.3389/fgene.2022.946869 (PMC9492296; doi:10.3389/fgene.2022.946869)
Supplement: Supplementary file 4 [file Table3.DOCX]

Supplementary Table 3: Number of SNP sites on each chromosome

| Choromosome | Number of sites |
| --- | --- |
| 1A | 202 |
| 1B | 264 |
| 1D | 140 |
| 2A | 243 |
| 2B | 298 |
| 2D | 165 |
| 3A | 234 |
| 3B | 289 |
| 3D | 278 |
| 4A | 129 |
| 4B | 106 |
| 4D | 133 |
| 5A | 219 |
| 5B | 245 |
| 5D | 126 |
| 6A | 128 |
| 6B | 212 |
| 6D | 246 |
| 7A | 228 |
| 7B | 199 |
| 7D | 333 |
| **Genome** | **Sites** |
| A | 1383 |
| B | 1613 |
| D | 1421 |
